# Exosomes as regenerative therapeutics for spinal cord injury: mechanisms and clinical prospects

**DOI:** 10.3389/fmed.2026.1810270

**Published:** 2026-04-20

**Authors:** Yingbo Xiao, Tao Cui, Ying Zhou, Qichao Su, Duan Yi

**Affiliations:** 1 Department of Pain Medicine, The Third Hospital of Qinhuangdao, Qinhuangdao, Hebei, China; 2 Department of Pain Medicine, Emergency General Hospital, Beijing, China; 3 Department of Anesthesiology, The Third Hospital of Qinhuangdao, Qinhuangdao, Hebei, China; 4 Department of Pain Medicine, Peking University Third Hospital, Beijing, China

**Keywords:** clinical prospect, exosome, neuroprotective effect, regenerative therapeutic, signal pathway, spinal cord injury

## Abstract

Exosomes are nanometer-scale extracellular vesicles secreted by cells with a diameter of approximately 30–100 nanometers. Serving as essential messengers for intercellular communication, they play significant roles in both physiological and pathological processes. Their low immunogenicity, excellent tissue penetrability, and high biocompatibility have positioned them as a research focus for disease diagnostic biomarkers and drug delivery vehicles. Spinal cord injury (SCI) is a severe traumatic disorder of the nervous system, often leading to neuronal death, axonal disruption, glial scar formation, and dysregulated inflammatory responses, ultimately resulting in irreversible sensory and motor dysfunction. This review systematically elucidates the pivotal roles of exosomes derived from various cell sources in the repair of SCI. It focuses on how these exosomes target key cellular components including neurons, glial cells, vascular endothelial cells, and immune cells. This interaction modulates core pathological processes such as neuroinflammation, glial scar formation, axonal regeneration, angiogenesis, and apoptosis. By synthesizing current evidence, we aim to unravel the complex regulatory networks mediated by exosomes as intercellular signaling hubs within the SCI microenvironment. Furthermore, this review provides a theoretical foundation for their future development as novel diagnostic tools, regenerative therapeutic vectors, and targeted intervention strategies for SCI.

## 1 Introduction

**FIGURE 1:**
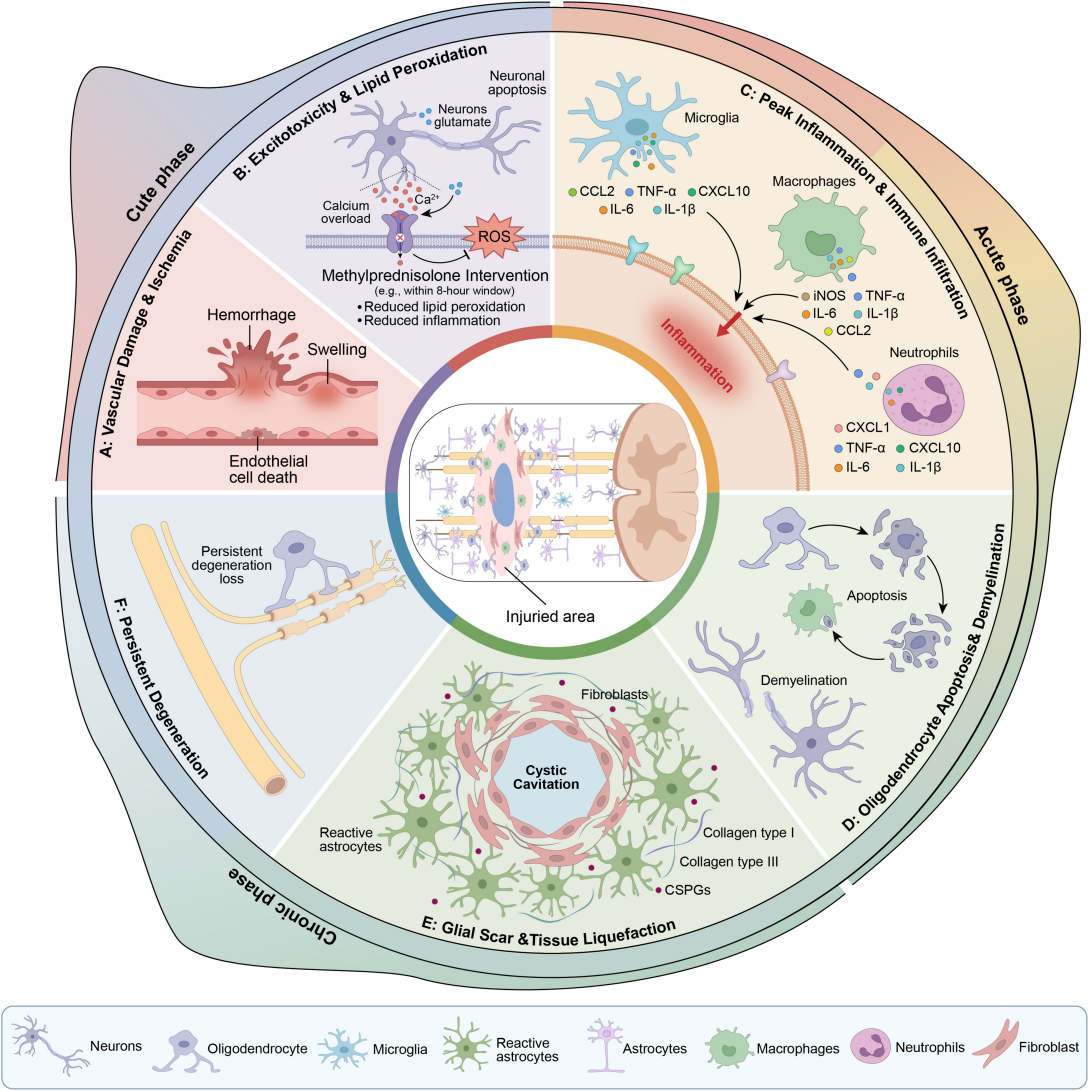
The three phases of pathological process regarding SCI.

Spinal cord injury (SCI) refers to a severe neurological trauma disorder caused by external forces, diseases, or degenerative changes, which leads to the destruction of the structure or function of the spinal cord, resulting in varying degrees of dysfunction in sensory, motor, and autonomic nerve functions below the injury site (1). Globally, the annual incidence of SCI is approximately 10–80 cases per 100,000 people, and its incidence varies depending on regions, economic levels, and trauma prevention measures (2). The causes of SCI can be mainly divided into two categories: traumatic and non-traumatic. The former includes external violence such as traffic accidents, falls from heights, violent incidents, and sports injuries, while the latter can be caused by tumor compression, vascular lesions, spinal infections, or degenerative changes (3). The pathophysiology of SCI unfolds as a dynamic cascade encompassing three temporal phases: acute, subacute, and chronic. The acute phase is marked by primary mechanical disruption followed by secondary injury events, including vascular rupture, ischemia, excitotoxicity, and oxidative stress. In the subacute phase, inflammation peaks with robust immune cell infiltration accompanied by oligodendrocyte apoptosis, demyelination, and tissue liquefaction. The chronic phase is characterized by astrogliosis and glial scar formation surrounding cystic cavities, along with persistent demyelination, Wallerian degeneration, and a hypoxic microenvironment (4–7). These processes interweave with each other, ultimately forming a pathological microenvironment that inhibits regeneration and is unfavorable for repair, as illustrated in Figure 1.

At present, the clinical treatment of SCI still faces significant challenges. The acute phase treatment mainly focuses on surgical decompression, stabilizing the spine, and high-dose methylprednisolone shock therapy, aiming to minimize secondary damage to the greatest extent (8). The rehabilitation treatment in the chronic phase is crucial for improving function and enhancing quality of life, but it cannot achieve fundamental regeneration of neural structures (9). Although emerging therapies such as stem cell transplantation, biomaterial scaffolds, and delivery of neurotrophic factors have shown potential in preclinical research, their clinical translation is still limited by bottlenecks such as immune rejection, tumor risk, low delivery efficiency, poor targeting, and high cost (10). Therefore, exploring new treatment strategies that can effectively regulate the pathological microenvironment after injury, promote endogenous repair, and be safe and efficient is the core frontier of current SCI research (11).

Exosomes are a type of nano-sized vesicles that are actively secreted into the extracellular space by cells after the fusion of intracellular multivesicular bodies with the cell membrane (12). Almost all types of cells can secrete exosomes, which are naturally present in various body fluids suchas blood, cerebrospinal fluid, and urine (12). Exosomes have a typical lipid bilayer membrane structure, and their membranes are rich in transmembrane protein families, heat shock proteins, and other characteristic proteins (13). More importantly, exosomes encapsulate the specific and complex biological activities of the source cells, enabling them to serve as key messengers for intercellular communication, transferring genetic and metabolic information from the source cells to distant or adjacent target cells, thereby regulating the physiological or pathological states of the latter (14). Additionally, as cell-free therapeutic agents, exosomes possesses a series of unique advantages, including low immunogenicity, biopassage ability, high biocompatibility and stability, and inherent targeting potential (15). Therefore, exosomes are regarded as a highly promising cell-free therapeutic platform, drug delivery carrier, and source of disease diagnostic markers (16).

In conclusion, the treatment of SCI urgently requires new strategies that can effectively intervene in its complex pathological microenvironment. With their unique biological properties, exosomes provide a novel perspective and tool for solving this problem. This review aims to deeply explore the exosomes from different cell sources, which regulate core pathological physiological processes such as neuroinflammation, cell apoptosis, glial scar formation, axonal regeneration, and angiogenesis through the specific active molecules they carry. We hope to provide a solid theoretical basis and a clear research framework for understanding the core role of exosomes in SCI repair and promoting their clinical translation and application through this review,.

## 2 Therapeutic potential of exosomes

**FIGURE 2:**
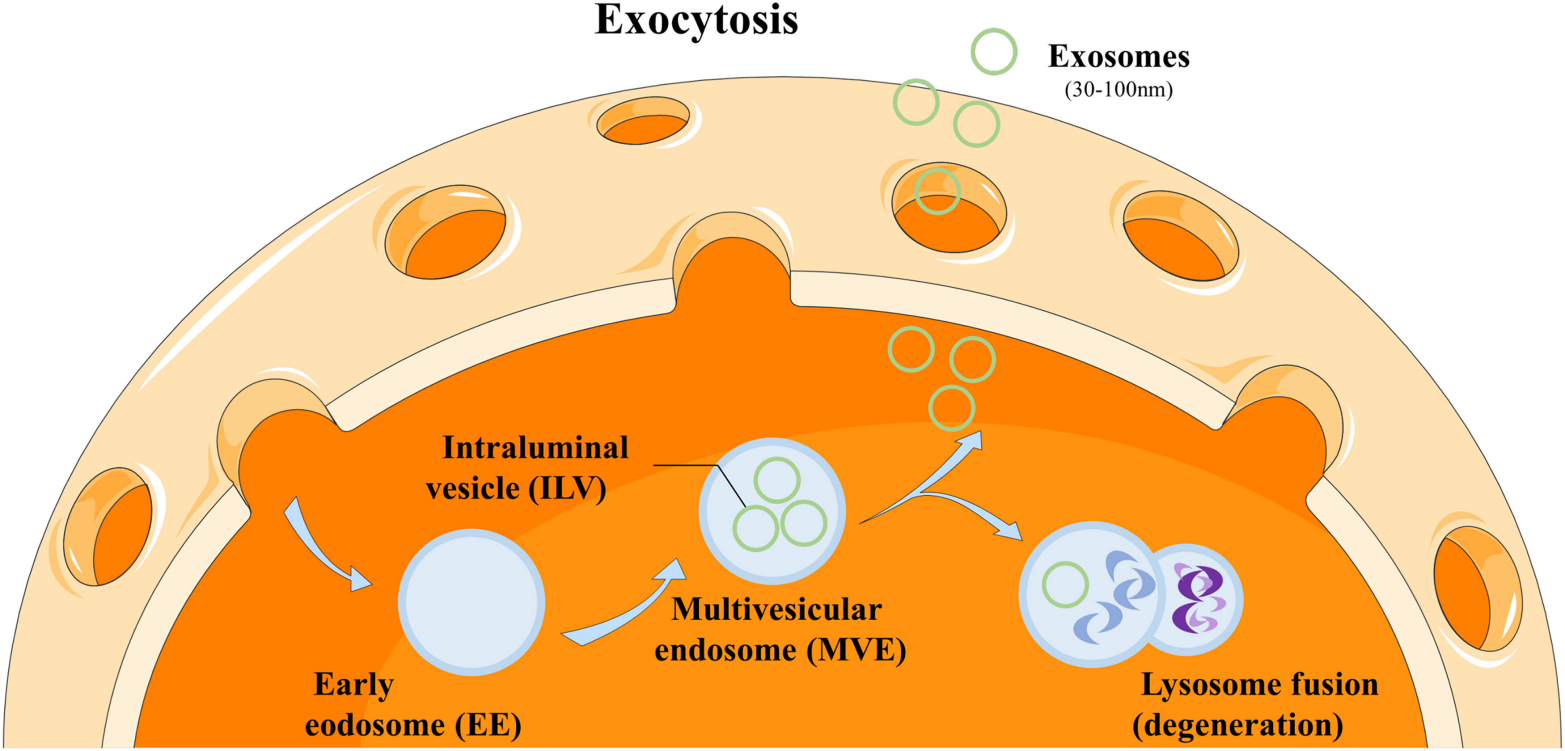
Production of exosomes.

Exosomes are a type of nanoscale extracellular vesicles secreted by cells, with a diameter of approximately 30–100 nanometers (17). They originate from the endocytic system within cells: the cell membrane invaginates to form early endosomes, which gradually evolve into multivesicular endosomes containing multiple intracellular vesicles, and finally are released to the extracellular space through exocytosis (18), as shown in Figure 2.

**FIGURE 3:**
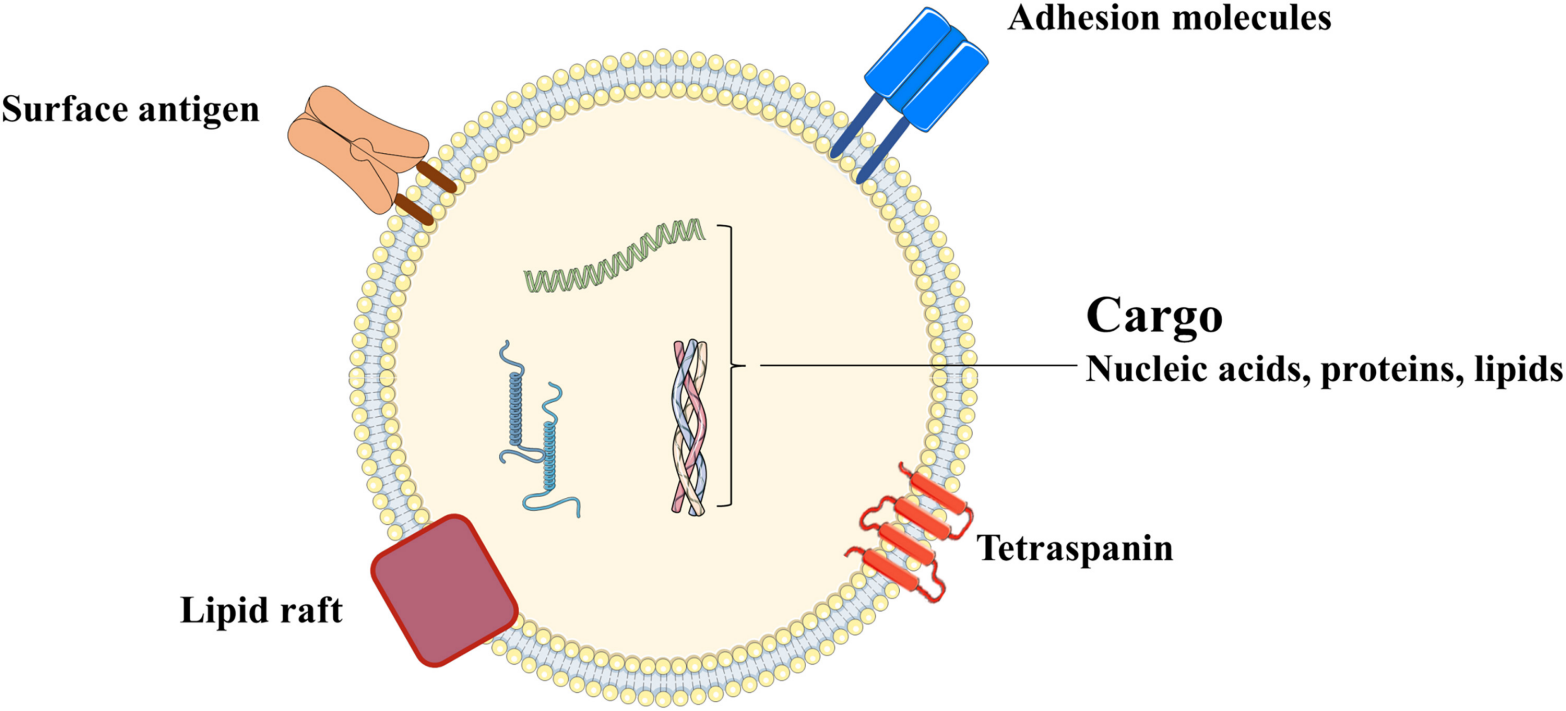
Structure of exosomes.

Exosomes possess a typical lipid bilayer membrane, with transmembrane proteins and lipid components mainly embedded on the membrane. The former includes CD9, CD63, CD81 and so on, while the latter includes cholesterol, sphingomyelin (SM), and phosphatidylserine (PS). Additionally, the interior of exosomes contains proteins, lipids, and various nucleic acids specific to the source cell, providing an important pathway for intercellular information transmission and material exchange (19), as shown in Figure 3.

**FIGURE 4:**
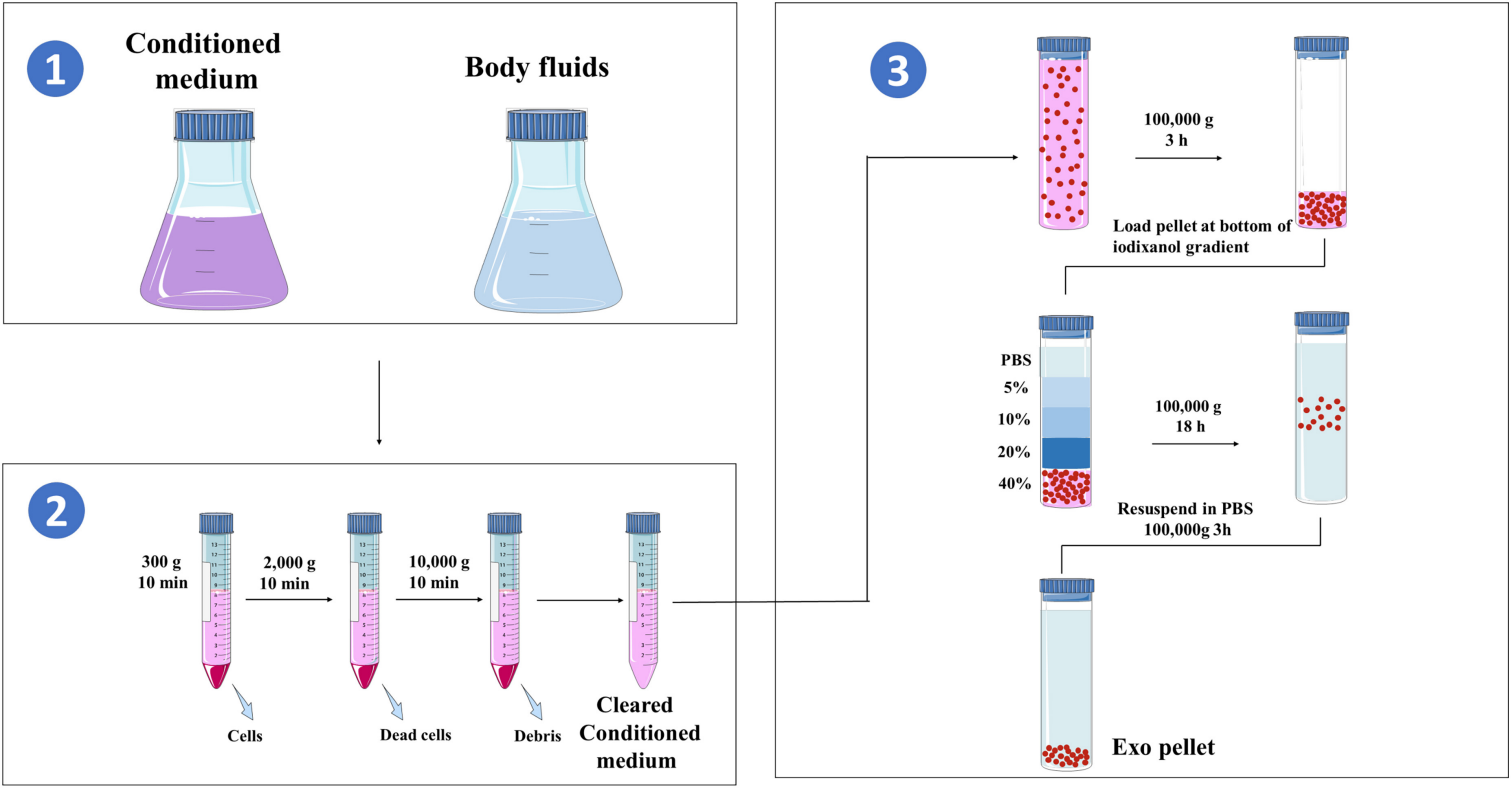
Purification of exosomes.

To obtain high-purity exosomes for research and application, differential and ultracentrifugation methods are commonly used for separation. First, samples are collected from cell culture supernatants or body fluids, and through a series of low-speed differential centrifugations, intact cells, cell debris, and apoptotic bodies are gradually removed. Subsequently, after ultracentrifugation, a pellet rich in exosomes can be obtained (20, 21). This standardized process is crucial for ensuring the reliability and reproducibility of exosome research, as shown in Figure 4.

To address the differences in centrifugal force, duration, rotor type, washing steps, and subsequent purification procedures among different laboratories, the Basic Information for Extracellular Vesicle Research (MISEV) guidelines formulated by the International Society for Extracellular Vesicles (ISEV) provide an important standardized framework (22, 23). These guidelines emphasize that the identification of exosomes should not rely on a single method, but must integrate multi-dimensional evidence, including size analysis, marker detection, and purity assessment. Therefore, we should use the MISEV guidelines as the main reference to evaluate the consistency of exosome preparation and characterization methods. This approach is of great significance for objectively assessing their therapeutic potential and promoting subsequent clinical translation.

In neurological diseases, exosomes, due to their low immunogenicity, excellent biological barrier penetration ability, and rich bioactive molecules contained, exhibit unique therapeutic potential (24). Studies have shown that exosomes can deliver specific functional molecules such as microRNAs to regulate the inflammatory response in the injured area, inhibit cell apoptosis, promote axon regeneration and angiogenesis, thereby improving the pathological microenvironment (25). These characteristics make them not only a new window for understanding disease mechanisms but also regarded as a promising cell-free therapeutic strategy and targeted drug delivery system (26). With the standardization of isolation and identification techniques and the in-depth elucidation of functional mechanisms, exosomes are expected to provide innovative solutions for the diagnosis and treatment of various diseases.

## 3 The source of exosomes in SCI

### 3.1 Mesenchymal stem cell

#### 3.1.1 Bone marrow mesenchymal stem cell

Bone marrow mesenchymal stem cells (BMSCs) are pluripotent stem cells derived from bone marrow, known for their self-renewal capacity and ability to differentiate into osteoblasts, chondrocytes, and adipocytes (27). In addition to their multi-lineage differentiation potential, BMSCs possess notable immunoregulatory functions, modulating immune responses through paracrine signaling and direct cell-to-cell interactions. Their accessibility, low immunogenicity, and tissue repair capabilities have made BMSCs a focal point of research in regenerative medicine and immunotherapy. These cells have already shown substantial therapeutic promise in clinical settings, including in the treatment of bone and joint diseases, hematopoietic disorders, and autoimmune conditions.

##### 3.1.1.1 Promote nerve repair and regeneration

BMSC-derived exosomes (BMSCs-Exos) form a robust neural repair network through multi-target and multi-pathway interactions (28). Their core mechanism involves directly activating the intrinsic regenerative program of neurons while inhibiting cell death (29). Certain exosome subpopulations, such as CD271^+^CD56^+^ BMSCs-Exos, carry molecules like miR-431-3p, which relieve the inhibitory effects of axon growth suppressors such as repulsive guidance molecule family member A (RGMA), thereby promoting axon elongation and branching (30). Additionally, they activate key survival and regenerative pathways, including Wingless-related integration site/beta-catenin (Wnt/β-catenin) and phosphatase and tensin homolog (PTEN)/protein kinase B (AKT)/mechanistic target of rapamycin (mTOR), facilitating neural regeneration (29, 31). BMSCs-Exos also prevent neuronal apoptosis by upregulating B-cell lymphoma 2 (Bcl-2), downregulating Bcl-2-associated X protein (Bax) and caspase-related proteins (28, 29), and utilizing miR-455-5p to target and inhibit myeloid differentiation primary response 88 (MyD88)/neurite outgrowth inhibitor-A (Nogo-A), inducing protective autophagy and further preventing apoptosis through multiple pathways (32). Furthermore, exosomes derived from BMSC pellets, rich in neurotrophic factors, guide neural stem cells (NSCs) toward neuronal differentiation, offering a new source for neural repair and synergistically promoting axon regeneration, synapse formation, and functional recovery (33, 34).

##### 3.1.1.2 Regulate inflammation and immune responses

BMSCs-Exos precisely regulate innate immune cells and key inflammatory pathways within the central nervous system (CNS), effectively counteracting the detrimental inflammatory microenvironment following SCI (35). Their actions focus on inhibiting the toxic activation of major glial cells: by targeting the toll-like receptor 4 (TLR4)/MyD88/Nogo-A/nuclear factor kappa-B (NF-κB) axis to reduce excessive microglial activation (36), and by delivering Zinc finger and BTB domain-containing protein 4 (ZBTB4) to inhibit inter-alpha-trypsin inhibitor heavy chain 3 (ITIH3) and prevent astrocytes from adopting a neurotoxic A1 phenotype (37). At the molecular level, miR-497-5p carried by BMSCs-Exos directly targets thioredoxin-interacting protein (TXNIP), inhibiting NOD-like receptor family pyrin domain containing 3 (NLRP3) inflammasome activation and reducing the release of pro-inflammatory factors like interleukin-1 beta (IL-1β) (38). Additionally, BMSCs-Exos downregulate the IL-17 signaling pathway, suppressing the inflammatory cascade from multiple angles (39), thereby fostering an anti-inflammatory environment conducive to nerve repair.

**TABLE 1:** The mechanism of exosomes derived BMSCs regarding SCI.

Source	Animal	Model	Administration	Modification	Dose	Time	Key mechanisms	Pathway	Referencess
BMSC	Rat	10 g, 12.5 mm, T10	TI	–	1 μg/μL	4 weeks	Autophagy activation	–	(28)
	Rat	10 g, 50 mm, T9-11	TI	–	200 μL	4 weeks	Anti-apoptosis	Wnt/β-catenin	(29)
	Mice	1 mm resection, T8	LI	miR-431-3p	10 μL	8 weeks	Axon regeneration	RGMA	(30)
	Rat	Vascular clamp, 30 s, T10	TI	miR-26a	200 μg	4 weeks	Axon regeneration Anti-astrogliosis Anti-autophagy	PTEN/AKT/mTOR	(31)
	Rat	Vascular clamp	IT	miR-455-5p	2 μg/μL	48 hours	Anti-apoptosis Autophagy activation	Nogo-A	(32)
	Rat	10 g, 12.5 mm, T10	LI	–	200 μg	4 weeks	Proliferation, migration Tube formation Anti-apoptosis Anti-inflammation Glial scar formation Axon regeneration Anti-astrocytes activation	–	(35)
	Mice	IH impactor device, 70 kdyn, T10	TP	–	20 μg	2 weeks	Inhibit microglial activation	TLR4/MyD88/NF-κB	(36)
	Mice	10 g, 12.5 mm; T10	LI	–	200 g	4 weeks	Anti-apoptosis	ZBTB4 and ITIH3	(37)
	Rat	10 g, 12.5 mm, T10	LI	miR-497-5p	200 μL	1 weeks	Anti-apoptosis Anti-inflammation Anti-oxidative stress	TXNIP/NLRP3	(38)
	Rat	10 g, 30 mm, T10	TI	–	120 μg	4 weeks	Anti-ferroptosis	IL-17	(39)
	Rat	Percussion device, 2 N	TI	miR-219-5p	200 μg	4 weeks	Anti-ferroptosis	UBE2Z/NRF2	(40)
	Rat	2 N, T10	TI	–	200 μg	4 weeks	Anti-apoptosis Attenuate BSCB leakage Anti-pyroptosis	–	(41)
	Rat	8 g, 60 mm, T10	TI	–	200 μg	3 weeks	Migration and tube formation Maintain BSCB integrity Anti-inflammation	VEGF	(42)
	Rat	IH impactor device, 120 kdyn, T10	SC	–	100 μg	2 weeks	Attenuate BSCB disruption	TIMP2/MMP	(43)
	Mice	5 g, 10 mm, T10	LI	–	200 μl	8 weeks	Promote macrophage engulfment Axon regeneration	–	(45)
	Rat	10 g, 25 mm, T10	LI	–	–	6 weeks	Promote neural differentiation Axon regeneration	–	(46)
GIT1-OV BMSC	Rat	10 g, 12.5 mm, T10	TI	–	200 μg	4 weeks	Glial scar formation Anti-neuroinflammation Anti-apoptosis Axon regeneration	–	(33)
NGF-OV BMSC	Mice	2 mm resection, T10	LI	–	–	8 weeks	NSC differentiation Axon regeneration	–	(34)
Shh-OV BMSC	Rat	2 N, T10	TI	–	200 μl	4 weeks	Neuronal regeneration	–	(44)

*TI, Tail intravenous; LI, Local injection; IT, Intrathecal; SC, Subcutaneous; TP, Transplant; OV, Overexpression; kdyn, kilodynes; IH, Infinite Horizon; Shh, Sonic hedgehog.

##### 3.1.1.3 Reduction of oxidative stress and programmed cell death

BMSCs-Exos exert a comprehensive protective effect against both ferroptosis and pyroptosis, two cell death processes linked to oxidative stress. In ferroptosis, they counteract its core biochemical features by reducing Fe^2+^, lipid peroxide malondialdehyde (MDA), and reactive oxygen species (ROS) levels, while elevating antioxidant glutathione (GSH) levels (39). Through downregulation of pro-ferroptotic protein Acyl-CoA synthetase long chain family member 4 (ACSL4) and upregulation of anti-ferroptotic factors such as glutathione peroxidase 4 (GPX4), they restore cellular antioxidant balance (39, 40). Notably, miR-219-5p carried by exosomes targets Ubiquitin conjugating enzyme E2 Z (UBE2Z) to stabilize and activate nuclear factor erythroid-related factor 2 (NRF2), forming a pivotal miR-219-5p/UBE2Z/NRF2 anti-ferroptosis axis. Additionally, BMSCs-Exos inhibit cell pyroptosis by suppressing Caspase-1 activation and IL-1β release, which is vital for maintaining blood-spinal cord barrier (BSCB) integrity, particularly protecting pericytes (41).

##### 3.1.1.4 Protecting the blood-spinal cord barrier and promoting angiogenesis

BMSCs-Exos repair the damaged nerve vascular unit through a dual mechanism: enhancing the BSCB and restoring blood supply in the injured area (35). The barrier protection mechanism is primarily driven by the tissue inhibitor of metalloproteinases 2 (TIMP2) protein carried by the exosomes, which inhibits matrix metalloproteinase (MMP) activity, preserving tight junction proteins of vascular endothelial cells and reducing leakage and edema. When combined with Strophanthin II A Sulfonic Acid Sodium (STS), this effect is further amplified (42, 43). In terms of angiogenesis, BMSCs-Exos themselves possess potent angiogenic capabilities, promoting endothelial cell proliferation, migration, and tube formation, thereby increasing microvascular density (35). By genetically engineering BMSCs to overexpress the Shh factor, functionalized exosomes are produced that more effectively activate the vascular endothelial growth factor (VEGF) signaling pathway, enhancing blood perfusion and nutrient supply at the injury site, thus supporting nerve regeneration (42, 44).

##### 3.1.1.5 Regulating glial scars and promoting the clearance of myelin debris

BMSCs-Exos also actively remodel the microenvironment to facilitate regeneration by modulating glial cell activity and enhancing debris clearance. They significantly suppress the excessive proliferation of reactive astrocytes and matrix deposition, reducing the formation of glial scars that obstruct nerve regeneration. This effect has been confirmed in various models, including those with G protein-coupled receptor kinase interactor 1 (GIT1) overexpression exosomes (31, 33, 42). Furthermore, BMSCs-Exos enhance macrophage phagocytic efficiency for myelin debris by upregulating the pattern recognition receptor macrophage receptor with collagenous structure (MARCO) on macrophage surfaces (45). Efficient clearance of inhibitory debris not only mitigates chronic inflammation but also removes chemical barriers hindering axon regeneration, thus promoting neural recovery and playing a critical role in structural repair (45).

##### 3.1.1.6 Engineering strategies and delivery system optimization

To enhance the therapeutic efficacy and clinical applicability of BMSCs-Exos, recent research has optimized strategies in two key areas: “source enhancement” and “precise delivery” (46). In content engineering, parent cells are modified to produce “enhanced” exosomes that overexpress specific functional molecules such as GIT1, miR-26a, and miR-219-5p (31, 44). Regarding delivery systems, to address the challenge of rapid exosome clearance from the body, biocompatible injectable hydrogels are employed as carriers (30, 46). By loading exosomes onto these hydrogels and implanting them at the injury site, a localized, sustained-release “drug depot” is created, enabling prolonged and continuous exosome release. This approach significantly increases their effective concentration and residence time at the lesion site, ultimately improving therapeutic outcomes for structural repair and functional recovery (46).

Based on above reports, recent years BMSCs-Exos mainly exert neuroprotective effects through surface engineering strategies and the combination of nanomaterials. The former involves modifying with targeting peptides such as RGD or RVG to significantly increase the accumulation of exosomes at the damaged spinal cord site. The latter involves being carried on injectable hydrogels, which can achieve local and sustained release of exosomes, thereby prolonging the therapeutic effect and enabling minimally invasive drug delivery, as detailed in Table 1. The therapeutic benefits of BMSC-Exos extend beyond SCI to a wide spectrum of neurological disorders, with conserved mechanisms involving neuroprotection, immunomodulation, and tissue regeneration. Various miRNAs in BMSC-Exos can not only inhibit neuroinflammation and promote microglial cells’ phagocytosis of amyloid plaques to treat Alzheimer’s Disease (AD) (47), but also protect dopaminergic neurons by delivering miR-133b and miR-124, promoting neuronal survival and inhibiting α-synuclein aggregation to treat Parkinson’s disease (48). In traumatic brain injury (TBI), they can also improve neurological function by inhibiting neuroinflammation and promoting neurogenesis, while reducing injury volume and enhancing cognitive function (49). Despite these advances, it is important to note that while numerous clinical trials are investigating BMSC-exos for various diseases, there are currently no commercially approved BMSC-Exos products neurological disorder on the market. Most products remain in early-phase clinical development, with ongoing efforts to address challenges in large-scale manufacturing, standardization, and regulatory approval.

#### 3.1.2 Umbilical cord mesenchymal stem cell

Umbilical cord mesenchymal stem cells (UCMSCs) are pluripotent stem cells isolated from the tissue of newborn umbilical cords, known for their high self-renewal capacity and multi-lineage differentiation potential (50). Compared to stem cells from other sources, UCMSCs offer advantages such as low immunogenicity, strong proliferative ability, and the absence of ethical concerns. They can also regulate immune responses, suppress inflammation, and promote tissue repair through paracrine effects (51). Currently, UCMSCs show significant potential for clinical application in fields like neurological disorders, autoimmune diseases, and tissue regeneration. Exosomes derived from UCMSCs (UCMSCs-Exos) intervene in multiple key pathological processes following SCI by delivering bioactive molecules such as miR-216, miR-149, and miR-223-5p. These exosomes effectively inhibit neuronal apoptosis and oxidative stress by modulating signaling pathways such as PTEN/AKT, phosphatidylinositol 3-kinase (PI3K)/AKT, and NRF2-Keap1. They also alleviate neuroinflammation by targeting the NLRP3 inflammasome and regulating M1/M2 microglial polarization. Additionally, UCMSCs-Exos enhance tight junction protein expression via the miR-501-5p/myosin light chain kinase (MLCK) and miR-149/endothelin (ET-1) axes, thereby stabilizing BSCB MyD88 function and fostering a favorable microenvironment for neural repair (52–56).

To further enhance the targeting and therapeutic efficacy of UCMSCs-derived exosomes, numerous cutting-edge engineering strategies have been explored. Xie, Fan, and colleagues specifically targeted exosomes by attaching arginine-glycine-aspartic acid (RGD) and rabies virus glycoprotein (RVG) peptides, enabling them to accumulate at damaged sites of new blood vessels or neurons (52, 57). Other researchers have directly loaded therapeutic molecules into exosomes using drugs or gene tools, creating “customized” exosomes with enhanced neuroprotective, anti-inflammatory, or angiogenic properties, including GPX4 activators, clustered regularly interspaced short palindromic repeats (CRISPR)/Caspase-9, and specific miRNA overexpression methods (53, 55, 57, 58). To optimize therapeutic outcomes, various intelligent delivery systems have been developed. For instance, biomimetic magnetoelectric hydrogels and thermosensitive injectable hydrogels have been utilized as controlled-release carriers for exosomes, enabling sustained release and synergistic physical therapy (54, 59). A more comprehensive approach involves combining engineered exosomes with NSC scaffolds for co-transplantation, which not only inhibits inflammation but also provides structural support, promoting nerve regeneration and angiogenesis. This strategy demonstrates potential for repairing complete SCI (60). These advanced delivery systems significantly enhance the precision, duration, and overall therapeutic effect.

hUCMSC-Exos primarily regulate the pathological microenvironment following SCI through paracrine effects. The various cytokines and exosomes they secrete can effectively reduce neuroinflammation, alleviate oxidative stress, and promote angiogenesis. Additionally, these exosomes provide structural support for axonal regeneration and myelin repair through direct differentiation or indirect supporting effects, thereby creating optimal conditions for spinal cord function recovery, as detailed in Table 2.

**TABLE 2:** The mechanism of exosomes derived UCMSCs regarding SCI.

Source	Animal	Model	Administration	Modification	Dose	Time	Key mechanisms	Pathway	Referencess
UCMSC	Mice	10 g, 2.5 cm, T10	IN	miR-501-5p	1 mg/kg	8 weeks	Reduce BSCB destruction	MLCK	(52)
	Rat	Vascular clamp, 60 s, T10	LI	miR-138-5p	–	4 weeks	Anti-oxidative stress Anti-inflammation Inhibit microglia polarization Axon regeneration	NLRP3-caspase1/Nrf2-keap1	(54)
	Mice	IH impactor device, 90 kdyn	–	miRNA-216	–	4 weeks	Promote angiogenesis activities Vascular regeneration Anti-apoptosis	PTEN/AKT	(55)
	Rat	10 g, 25 mm, T10	TI	miR-149	200 μg	4 weeks	Anti-apoptosis Reduce BSCB leakage	PI3K/AKT	(56)
	Mice	5 g, 12.5 mm, T10	TI	–	1 mg/kg	2 weeks	Anti-ferroptosis Anti-inflammation Neural regeneration Reduce astrogliosis	GPX4	(57)
	Mice	10 g, 6.25 mm, T11-12	TI	–	50 μg	6 weeks	Anti-inflammatory Regulate macrophage polarization	TNF-α/NF-κB	(58)
	Rat	4 mm resection, T9-10	LI	–	200 μg	8 weeks	Anti-inflammatory Promote NSCs differentiation Axon regeneration	PI3K-AKT	(59)
	Rat	3-10 mm resection, T9-11	TP	–	–	4 weeks	Regulate microglia polarization Promote cellular regeneration	–	(60)
Tanshinone- UCMSC	Mice	0.6 mm impactor	TI	miR-223–5p	–	4 weeks	Anti-inflammation Regulate immune microenvironment	USP8/NLRP3	(53)

*IN, Intranasal;USP8, Ubiquitin specific peptidase 8.

#### 3.1.3 Adipose-derived stem cell

Adipose-derived stem cells (ADSCs) are versatile adult stem cells sourced from adipose tissue, known for their ability to secrete protective factors and differentiate into neural cells. In the nervous system, ADSCs primarily facilitate repair by reducing inflammation, supporting neuron survival, and promoting regeneration, offering significant potential for treating various conditions (61).

Ferroptosis in SCI leads to the programmed death of neurons and glial cells through the abnormal accumulation of lipid peroxides and the imbalance of the GSH antioxidant system, exacerbating secondary neurodegeneration after the primary injury. This process not only compromises cell membrane integrity but also triggers persistent inflammation and dysfunction of the BSCB, hindering nerve regeneration and functional recovery. Ferroptosis has thus become a key mechanism in the pathological progression of SCI. Several studies have demonstrated that exosomes derived from ADSCs (ADSCs-Exos) can inhibit ferroptosis by activating the NRF2- solute carrier family 7 Member 11 (SLC7A11)-GPX4 pathway, promoting the recovery of vascular and neural functions both *in vivo* and *in vitro* (62). Additionally, ADSCs-Exos suppress the expression of ROS and inflammatory cytokines via the circular Wdfy3 (63). Bioinformatics analysis has revealed that GPX4 and miR-423-3p are downstream targets of circular WD repeat and FYVE domain containing 3 (Wdfy3), and upregulation of GPX4 or downregulation of miR-423-3p effectively contributes to SCI functional repair.

Shao et al. have confirmed that ADSCs-Exos containing the ring structure of Astn1 protect BV2 cells by targeting the miR-138-5p/autophagy related 7 (Atg7) pathway. Luciferase reporter assays verified that miR-138-5p and Atg7 are downstream targets of ring-Astn1. This therapeutic effect occurs through autophagy activation induced by the upregulation of ring-Astn1 (64). In another study, ADSCs were pre-treated with hypoxia before exosome extraction. The results showed that hypoxic exosomes (Hexos) promoted the expression of peroxisome proliferator-activated receptor γ (PPARγ) by inhibiting miR-130b-3p, thereby facilitating the M1-to-M2 microglial polarization. Luciferase reporter assays further confirmed that lncGm37494 plays a pivotal role in regulating the microenvironment (65). Another study highlighted the critical role of farnesyl-diphosphate farnesyltransferase 1 (FDFT1) in SCI repair. Bioinformatics analysis and gene expression profiles from inflammatory cell models revealed that overexpression of FDFT1 significantly reversed the inflammatory response and apoptosis of BV2 cells induced by heme. Mechanistically, ADSCs-Exos influence FDFT1 expression through the ceRNA mechanism mediated by insulin-like growth factor 2 mRNA-binding protein 2 (IGF2BP2), thereby inhibiting inflammation (66).

ADSCs-Exos exhibit a unique triple therapeutic potential for SCI. They not only effectively inhibit neuroinflammation and mitigate secondary damage by delivering bioactive substances that create a supportive microenvironment for neural repair, but also activate endogenous neural regeneration mechanisms, promoting axon growth and myelin remodeling. This multi-targeted approach significantly enhances neural functional remodeling, providing innovative therapeutic strategies for achieving both structural and functional repair in SCI, as detailed in Table 3.

**TABLE 3:** The mechanism of exosomes derived ADSCs regarding SCI.

Source	Animal	Model	Administration	Modification	Dose	Time	Key mechanisms	Pathway	References
ADSC	Rats	2 millimeters, T10	TI	–	100 μg	2 weeks	Anti-oxidative stress Anti-ferroptosis	NRF2/SLC7A11/ GPX4	(62)
	Mice	10 g, 6.5 cm, T10	TI	miR-138-5p	200 μg	4 weeks	Anti-inflammation Anti-ferroptosis Anti-apoptosis Anti-oxidative stress	circ-Wdfy3/GPX4	(63)
	Mice	5 g, 6.5 cm, T10	TI	miR-138-5p	200 μg	4 weeks	Autophagy activation	circ-Astn1/Atg7	(64)
	Rat	10 g, 25 mm, T10	TI	miR-130b-3p	200 μg	4 weeks	Anti-inflammation	lncGm37494	(65)
	–	–	–	–	–	–	Anti-inflammation Anti-apoptosis	FDFT1	(66)

*Adipose-derivedstemcell (ADSC).

#### 3.1.4 Other stem cell

Human placental mesenchymal stem cells (hPMSCs) are multipotent stromal cells derived from the placental mesoderm, characterized by robust self-renewal capacity and the ability to differentiate into various cell lineages. They exhibit significant immunomodulatory properties and secrete a range of trophic factors, making them a promising candidate for regenerative medicine and tissue repair applications (67). Numerous studies have demonstrated that exosomes derived from hPMSCs (hPMSCs-Exos) can effectively promote angiogenesis and neurological recovery. *In vitro*, hPMSCs-Exos enhance tube formation and migration of human umbilical vein endothelial cells (HUVECs), while *in vivo*, they lead to significant increases in vascular density, volume fraction, and connectivity, alongside notable improvements in motor and sensory functions. Further research indicates that hPMSCs-Exos exhibit synergistic neuroprotective effects when combined with hyperbaric oxygen (HBO) therapy, significantly improving neurological function scores, neuronal density, antioxidant factors, and the anti-inflammatory cytokine IL-10 (68–70).

Exosomes derived from dental pulp stem cells (DPSCs-Exos) demonstrate similar reparative functions: *in vitro*, they promote axonal growth, inhibit neuroinflammation and apoptosis, reduce glial hyperplasia, and significantly enhance neurological and motor function (71). In 2026, Wang et al. first revealed that exosomes derived from olfactory mucosa mesenchymal stem cells (OM-MSCs-Exos) promote axonal regeneration and functional recovery after SCI by delivering long non-coding RNA component of mitochondrial RNA processing endoribonuclease (RMRP). The mechanism involves RMRP binding to WT1 associated protein (WTAP) in astrocytes, reducing the expression of WTAP and the m6A modification of p53 mRNA, ultimately fostering a supportive microenvironment for neuronal axonal regeneration. This discovery sheds light on how OM-MSCs-Exos regulate glial cell metabolism and enhance neural repair through the RMRP/WTAP/p53 axis (72).

These findings highlight the potential of exosomes from various stem cell sources as promising therapeutic strategies for SCI, operating through multiple mechanisms such as promoting angiogenesis, modulating inflammation and oxidative stress, inhibiting apoptosis, and supporting axonal regeneration, as detailed in Table 4.

**TABLE 4:** The mechanism of exosomes derived other stem cells regarding SCI.

Source	Animal	Model	Administration	Modification	Dose	Time	Key mechanisms	Pathway	References
hPMSC	Rat	Vascular clamp	TI	–	0.1 μg/μL	48 h	Anti-apoptosis Anti-gliosis Anti-oxidative stress Anti-inflammation	–	(68)
	Mice	Modified Allen’s apparatus	LI	–	–	6 weeks	Tube formation and migration Angiogenesis	–	(69)
MSC	Mice	5 g, 6.5 cm, T10	TI	miR-216a-5p	200 μL	4 weeks	Promote M2 polarization	TLR4/NF-κB/PI3K/AKT	(70)
hDPSC	Mice	IH impactor device, 70 kdyn, T10	IT	–	20 μL	4 weeks	Axon growth Anti-apoptosis Anti-inflammation	–	(71)
OM-MSC	Mice	8 g, 60 mm, T10	TI	–	200 μg	4 weeks	Axon regeneration	WTAP-mediated p53 m6A	(72)

*Human placental mesenchymal stem cell (hPMSC); Mesenchymal stem cell (MSC); Dental pulp stem cell (hDPSC); Olfactory mucosa mesenchymal stem cell (OM-MSC).

### 3.2 Nerve cell

#### 3.2.1 Nerve stem cell

NSCs in the spinal cord are a unique population of self-renewing, multipotent cells with intrinsic potential for neural regeneration and repair, acting as a key cellular reservoir in the CNS (73). Enriched with VEGF-A, NSC-derived exosomes (NSC-Exos) directly enhance vascular regeneration by stimulating angiogenic activity in spinal cord microvascular endothelial cells (SCMECs), accelerating microvascular regeneration, reducing lesion cavities, and improving functional outcomes in SCI mice (74). Furthermore, the combination of NSC-Exos with therapeutic agents shows synergistic effects, enhancing barrier protection for SCMECs under hypoxic conditions and reducing apoptosis, potentially through modulation of the PTEN/AKT pathway (75). These studies highlight that NSC-Exos function as sophisticated, multi-functional nanotherapeutics. They orchestrate repair by simultaneously targeting and integrating key pathological processes—neuroinflammation, vascular disruption, and multiple forms of neuronal cell death, through the delivery of specific proteins and miRNAs (76). Notably, NSC-Exos also provide direct neuroprotection by inhibiting distinct programmed cell death pathways. They suppress neuronal necroptosis by disrupting the receptor-interacting protein kinase 1 (RIPK1)-RIPK3 interaction and mitigate ferroptosis via the delivery of miR-218a-5p, which triggers a cascade involving B cell-specific Moloney murine leukemia virus integration site 1 (Bmi1)-mediated methyltransferase-like 3 (Mettl 3) degradation, a reduction in ribosomal protein S6 kinase beta-1 (p70S6K) mRNA, and ultimately, decreased lipid peroxidation (77). Additionally, Liu et al. demonstrated that exosomes derived from induced pluripotent stem cell-derived NSCs (iPSC-NSCs-Exos) via let-7b-5p alleviated microglial and macrophage pyroptosis and reduced secondary inflammation by targeting leucine-rich repeats and immunoglobulin-like domains protein 3 (LRIG3), thereby promoting motor recovery (78). Chen et al. reported that epidermal growth factor receptor-positive NSCs (EGFR^+^ NSCs), highly enriched with miR-34a-5p, were internalized by neurons to inhibit histone deacetylase 6 (HDAC6) expression, promoting microtubule stabilization and inducing autophagy, which further supports structural and functional recovery after SCI (79).

NSC-Exos represent a promising cell-free therapeutic platform for SCI, offering targeted delivery of neuroprotective and regenerative molecules while mitigating the risks associated with whole-cell transplantation. Their ability to modulate multiple pathological processes, such as neuroinflammation, vascular dysfunction, and programmed neuronal death, positions them as versatile candidates for future combinatory and precision medicine strategies in neural repair, as detailed in Table 5.

**TABLE 5:** The mechanism of exosomes derived NSCs regarding SCI.

Source	Animal	Model	Administration	Modification	Dose	Time	Key mechanisms	Pathway	References
NSC	Mice	8 g, 3 cm, T10	TI	–	200 μg	4 weeks	Angiogenic activities Microvascular regeneration	VEGF-A	(74)
	Rat	3 cm, T10	TI	miR-218a-5p	100 μg	4 weeks	Anti-ferroptosis Ubiquitination and degradation	Bmil/Mettl3/Alox12	(75)
	Mice	10 g, 4 cm, T10	EPI and IV	–	–	2 weeks	Anti-neuronal necroptosis	RIPK1-RIPK3	(77)
	Rat	50 mm contusion, T10	TI	–	20 μg	4 weeks	Anti-apoptosis	PTEN/AKT	(79)
EGFR-NSC	Mice	10 g, 2.5 cm, T10	LI	miR-34a-5p	10 mg/mL	4 weeks	Neural regrowth Microtubule stabilization Autophagy Activation	HDAC6	(76)
iPSC-NSC	Mice	5 g, 6.5 cm, T8	IT	let-7b-5p	20 μg/μL	4 weeks	Anti-pyroptosis	LRIG3	(78)

*EPI, Epidural injection; IV, Intravenous injection.

#### 3.2.2 Other nerve cells

The complex pathological microenvironment following SCI presents substantial challenges for neural repair. However, exosomes derived from various glial cells and Schwann cells, which serve as key mediators of intercellular communication, exhibit multifaceted therapeutic potential in modulating this microenvironment. Microglia, the immune cells of the CNS, play a critical role in responding to stimuli to protect neuronal cells from death. Li et al. confirmed that microglia-derived exosomes exert neuroprotective effects by enriching miR-151-3p, which targets p53 and regulates the p21/cyclin-dependent kinase 1 (CDK1) signaling pathway, thereby inhibiting neuronal apoptosis and promoting axonal regeneration (80).

Additionally, exosomes secreted by M2-type microglia not only inhibit the melanoma deficiency gene 2 (AIM2)/apoptosis-associated speck-like protein (ASC)/caspase-1 signaling pathway by carrying miR-672-5p, thereby reducing neuronal pyroptosis (81), but also stimulate the growth of dorsal root ganglion NSCs and axons, regulate M2 microglial polarization (82), promote neuronal survival and axon preservation, reduce lesion areas, inhibit A1 astrocyte activation, and improve motor function recovery in SCI mice (83). This effect is mediated by the inhibition of the NF-κB signaling pathway. These mechanisms make M2-derived exosomes (M2-Exos) a promising therapeutic strategy for SCI.

In parallel, Cui et al. reported that resveratrol-loaded microglia-derived exosomes (R-MDEs) effectively accumulate at the injury site, reprogram astrocyte metabolism, inhibit ROS production, and reduce glial scar formation, thereby achieving synergistic anti-inflammatory and neuroprotective effects (84). Beyond microglia, exosomes derived from A2-type astrocytes (A2-Exos) have shown potential in promoting motor function recovery and BSCB repair. This effect is attributed to the enrichment of miR-5121, which targets AKT2 and regulates the AKT/mTOR/p70S6K pathway, enhancing autophagy in endothelial cells (85). In 2025, Singh et.al first reported that exosomed derived from glial cells also could excert neuroprotective effect through promoting the growth of axons and inhibiting the formation of glial scars via PTEN/AKT pathway (86). Furthermore, Schwann cell-derived exosomes (SCs-Exos) primarily promote the proliferation, migration, and tube formation of vascular endothelial cells by delivering integrin-β1, thereby enhancing angiogenesis, reducing tissue damage, and improving neurological functional recovery after SCI (87).

In summary, exosomes from various nerve cell sources contribute to post-SCI repair by carrying specific miRNAs or proteins that act on distinct cellular targets and signaling pathways. They synergistically promote BSCB repair, modulate immune inflammation, inhibit cell death, enhance angiogenesis, and support axonal regeneration, providing a solid theoretical foundation for developing cell-free therapeutic strategies based on exosomes, as detailed in Table 6.

**TABLE 6:** The mechanism of exosomes derived other nerve cells regarding SCI.

Source	Animal	Model	Administration	Modification	Dose	Time	Key mechanisms	Pathway	References
Microglia	Mice	10 g, 25 mm, T10	TI	miR-151-3p	200 μg	8 weeks	Axon growth Anti-apoptosis	p53/p21/CDK1	(80)
	Mice	10 g, 25 mm, T10	TI	miR-672-5p	200 μg	4 weeks	Axon regeneration Anti-pyroptosis	AIM2/ASC/Caspase-1	(81)
	Rat	Resection, T8-T10	TP	–	200 μg	8 weeks	Axon outgrowth Promote neurogenesis Axon regeneration and remyelination	PTEN/PI3K/AKT/ mTOR	(82)
	Mice	Crush with fine forceps	IV	–	200 μg	4 weeks	Promote neuronal survival Axon preservation Diminish scar formation and lesion area Astrocyte activation	NF-κB	(83)
	Mice	10 g, 25 mm, T10	TI	–	–	4 weeks	Anti-oxidative stress	–	(84)
A2 astrocyte	Mice	5 g, 6.5 cm, T10	TI	miR-5121	200 μg	4 weeks	Reconstruct BSCB Autophagy activation	AKT2/mTOR/ p70S6K	(85)
C6 glial cells	Rat	10 g, 10 cm, T10-12	IV	Rab27a	25 ng	2 weeks	Axon growth Inhibit glial scars	PTEN/AKT	(86)
SC	Rat	8 g, 4 cm, T10	TI	–	100 μg	4 weeks	Promote angiogenesis	Integrin-β1	(87)

*Schwann cell (SC).

### 3.3 Immune cell

#### 3.3.1 Macrophage

Macrophages are essential immune regulators, continuously monitoring the microenvironment to maintain neural homeostasis. Their dynamic functions, including debris clearance, inflammatory regulation, and tissue repair, position them as pivotal players in both the health and disease states of the CNS (88). Following SCI, the proportion of M2 macrophages significantly decreases. Exosomes derived from M2 macrophages can promote M2 polarization and inhibit the M1 phenotype, while exosomes from M1 macrophages have the opposite effect. This phenotypic transformation is likely mediated by the miRNA-mRNA network within exosomes, with the miR-23a-3p/PTEN/PI3K/AKT axis playing a pivotal role. These findings suggest that macrophage polarization can be modulated by their own exosomes, with M2 exosomes having the potential to promote M2 polarization through this network (89). Moreover, M2 exosomes can inhibit ubiquitination through OTU deubiquitinase (OTULIN), activate the Wnt/β-catenin signaling pathway, and enhance angiogenic activity in spinal microvascular endothelial cells. When released through hydrogel, they significantly promote vascular regeneration and functional recovery in SCI mice (90). Given the increased expression of chemokine receptors after SCI, Fu et al. engineered a viral macrophage inflammatory protein II-lysosome-associated membrane protein 2b-gene-modified M2 exosome (vMIP-II-Lamp2b-M2-Exo). vMIP-II, a broad-spectrum chemokine receptor-binding peptide, and Lamp2b, a key membrane component of exosomes, enabled targeted delivery of M2 exosomes to the damaged spinal cord. The results showed that vMIP-II-Lamp2b-M2-Exo provided neuroprotection by targeting the injured area, inhibiting chemokine signaling, reducing pro-inflammatory factor production, and regulating microglia/macrophage polarization (91). In 2021, Zhang et al. reported the critical role of peripheral macrophage-derived exosomes (PM-Exos) in SCI. They demonstrated that PM-Exos play a significant role in local microglial signaling via intravenous injection. By inhibiting the PI3K/AKT/mTOR signaling pathway, PM-Exos activated microglial autophagy and enhanced anti-inflammatory microglial polarization (92).

Exosomes derived from macrophages, particularly those engineered from peripheral macrophages, regulate neuroinflammation, promote angiogenesis, and activate microglial autophagy by delivering specific miRNAs and proteins. These effects illustrate the significant therapeutic potential of macrophage-derived exosomes in neural repair after SCI, as detailed in Table 7.

**TABLE 7:** The mechanism of exosomes derived Microglias regarding SCI.

Source	Animal	Model	Administration	Modification	Dose	Time	Key mechanisms	Pathway	References
Macrophage	Rat	2.5 mm diameter rob,2.5 cm, T10	TI	miR-23a-3p	200 μl	–	Modulate polarization	PTEN/PI3K/AKT	(89)
	Mice	10 g, 20 mm, T10	LI	–	100 μl	4 weeks	Angiogenic activities Vascular regeneration	Wnt/β-catenin	(90)
	Mice	1.3 mm diameter, 50 Kdyn, T9	TP	–	10μg	6 weeks	Modulate polarization Anti-inflammation	AKT	(91)
PM	Rat	10 g, 3 cm, T10	TI	–	20/200 μg	1 weeks	Anti-inflammation Autophagy activation	PI3K/AKT/mTOR	(92)

* Schwann cell (SC).

#### 3.3.2 Endothelial cell

Emerging evidence highlights the key role of exosome-mediated intercellular communication in modulating macrophage/microglial polarization and functional recovery after SCI. Yuan et al. reported that endothelial progenitor cell-derived exosomes (Eps-Exos), carrying miR-222-3p, can shift microglia and macrophages from the M1 to the M2-like phenotype *in vitro* and improve motor function *in vivo*. This effect occurs primarily through inhibition of suppressor of cytokine signaling 3 (SOCS3) and activation of the janus kinase 2 (JAK2)/signal transducers and activators of transcription (STAT3) pathway (93). Similarly, exosomes from microvascular endothelial cells (MVECs) promote M2 polarization and alleviate mitochondrial damage, partly through the transfer of ubiquitin-specific protease 13 (USP13), which stabilizes inhibitor of nuclear factor kappa B α (IκBα) and suppresses the NF-κB signaling pathway (94).

These findings highlight how exosomal cargo can regulate key inflammatory pathways to drive anti-inflammatory polarization, offering a promising therapeutic strategy for spinal cord repair, as detailed in Table 8.

**TABLE 8:** The mechanism of exosomes derived endothelial cells and other sources regarding SCI.

Source	Animal	Model	Administration	Modification	Dose	Time	Key mechanisms	Pathway	References
EPC	Mice	10 g, 2 cm, T10	TI	miR-222-3p	100 μL	8 weeks	Anti-inflammatory	SOCS3/JAK2/STAT3	(93)
MVEC	Mice	5 g, 6.5 cm, T10	TI	–	200 μg	4 weeks	Rugulate polarization Ameliorate mitochondrial impairment	USP13-IκBα/NFκB	(94)
PRP	Mouse	Vascular clamp, 30 s, T9	LI	–	160 μg/mL	4 weeks	Anti-inflammation Enhance BSCB integrity	NF-κB	(95)
Peripheral	Rat	10 g, 12.5 mm, T10	LI	–	200 μg	4 weeks	Anti-apoptosis Anti-oxidative stress	–	(96)
Plasma	Rat	10 g, 12.5 mm, T10	LI	miR-138-5p	–	4 weeks	Reprogram microglia polarization	SOX4	(97)
SCI tissue	Rat	10 g, 2.5 cm, T10	IT	miR-155-5p	1 × 10^10^ particles	1 weeks	Exacerbate inflammation and injury	FoxO3a/NF-κB	(98)
Flos Sophorae	Rat	4 mm resection	LI	–	–	4 weeks	Anti-oxidative stress Promote nervous restoration ability	–	(99)

*SOX4, Sex-determining region Y-box 4.

### 3.4 Other sources

Platelet- or plasma-derived exosomes present a promising therapeutic strategy for SCI by stabilizing the BSCB, modulating neuroinflammation, and promoting neuroregeneration. Nie et al. explored an innovative approach using platelet-rich plasma-derived exosomes (PRP-Exos) to stabilize BSCB function and alleviate neuroinflammation. PRP-Exos were shown to reduce endothelial permeability, restore tight junction integrity, and mitigate neuroinflammation through the NF-κB signaling pathway. In an SCI model, local delivery of hydrogel-coated PRP-Exos reduced vascular leakage, enhanced tight junction protein expression, improved the inflammatory microenvironment, and promoted functional recovery (95). Similarly, two other studies highlighted the critical role of exosomes derived from peripheral blood in SCI. On one hand, they significantly reduced gliosis, cellular pyknosis, and neuronal swelling, while suppressing apoptosis and oxidative stress (96). On the other hand, they regulated microglial polarization to enhance anti-inflammatory capacities, attributed to the elevated levels of miR-138-5p (97).

Additionally, Xie et al. observed differences in the morphology, concentration, and function of exosomes derived from damaged and normal spinal cord tissues. Exosome miRNA sequencing combined with experimental validation revealed higher levels of miR-155-5p in SCI-Exos, leading to significant inhibition of forkhead box O3a (FoxO3a) phosphorylation and activation of the NF-κB pathway. This cascade promoted M1 microglial polarization and the expression of inflammatory cytokines. These findings suggest that targeting miR-155-5p expression or exosome secretion could be a novel strategy for alleviating inflammation and reducing secondary damage after SCI (98). In recent years, plant-derived exosomes have gained significant attention from researchers. For example, exosomes derived from Flos Sophorae Immaturus were shown to accumulate continuously in the lesion area, inhibit neuronal apoptosis, and regulate the microenvironment, resulting in significant neural function restoration (99).

These studies highlight the substantial therapeutic potential of exosomes from various sources in treating SCI. By enhancing barrier integrity, modulating neuroinflammation, and promoting neural repair, exosomes offer innovative targets and strategies for mitigating secondary damage and improving functional recovery, as detailed in Table 8.

## 4 Limitations and prospects

Although exosomes show significant potential in the repair of SCI, their clinical translation still faces multiple challenges. Firstly, the significant heterogeneity of exosomes severely impacts experimental reproducibility. Their biological activity is highly dependent on source cell type, culture conditions, and isolation methods. Currently, isolation techniques vary considerably across laboratories, leading to inconsistencies in exosome size, cargo, and function. While some studies adhere to MISEV guidelines with multi-dimensional characterization, others lack systematic identification. This heterogeneity hinders replication of therapeutic outcomes and introduces uncertainty in efficacy and safety assessment. Future research should rigorously follow MISEV guidelines to establish standardized protocols from source cell culture to multi-dimensional characterization. Engineering strategies can generate “functionally enhanced” exosomes, reducing dependence on native complex cargo and mitigating heterogeneity-related uncertainty. Additionally, developing exosome reference materials for cross-laboratory validation will be critical for advancing pharmaceutical translation.

Secondly, the targeted delivery efficiency of exosomes requires improvement. Although exosomes possess inherent homing capabilities, their in vivo delivery is hampered by low efficiency and insufficient specificity. The systemic administration not only limits therapeutic efficacy but also raises the risk of off-target adverse effects. The tail vein injection represents the most widely used systemic approach, offering advantages such as ease of operation and feasibility for repeated dosing. However, this route is constrained by rapid systemic clearance and the requirement to traverse the BSCB. In contrast, local delivery strategies, including intrathecal and direct local injection, bypass the BSCB and substantially enhance local bioavailability. Nevertheless, these invasive techniques are technically demanding and poorly suited for long-term repeated administration. Biomaterial-based sustained release systems have emerged as a compelling alternative. Future research could consider integrating exosomes with other emerging regenerative strategies. For instance, the “Nano-shields” concept proposed by Xie et al. (100) which utilizes nanoparticles with the activity of mimicking antioxidant enzymes, provides another powerful tool for directly removing excessive ROS generated in damaged areas, which is worthy of in-depth exploration in future combined treatment strategies. Furthermore, we can not only design exosomes using some professional methods to separate them from the “total exosomes,” but also identify the subgroups with specific characteristics and high efficiency. Moreover, they can precisely act on a certain pathological stage of SCI because of its dynamics, which helps to make them more clinically significant.

Thirdly, the majority of studies on exosome-based treatments for SCI are still limited to rodent models, which models have significant differences from humans in terms of spinal size, neural anatomy, immune response, and the repair process after injury, making it difficult to fully simulate the clinical situation. These data are essential for evaluating the safety, efficacy, and pharmacokinetics of exosome therapies. Future research should actively focus on preclinical evaluations using large animal models, such as pigs, dogs, and non-human primates, systematically assessing the safety, pharmacokinetic characteristics, and therapeutic effects of exosome therapies under more human-like anatomical and physiological conditions. These data will not only help optimize the dosing regimen but also provide key references for subsequent clinical trial design. From previous studies, we know that the most commonly used dose of exosomes in rats or mice is 200 micrograms. However, for humans, the conversion of this dose is not a simple linear relationship. The pharmacokinetics of exosomes in the complex human spinal cord environment remains a major obstacle in clinical trial design.

Fourthly, the clinical-scale production of exosomes presents formidable challenges. Good manufacturing practice (GMP)-grade manufacturing requires standardized cell culture systems, scalable isolation processes, and rigorous quality control. Additionally, the long-term stability of exosome formulations remains inadequately addressed, directly impacting their efficacy and safety during storage, transportation, and administration. These barriers must be overcome for exosome-based therapies to transition from laboratory to clinical practice. Accelerated efforts are needed to advance industrial-scale production and formulation development. Scalable isolation technologies compatible with GMP requirements should be established, alongside formulation strategies ensuring batch-to-batch consistency and long-term stability. Overcoming these technological bottlenecks is essential for exosome-based therapies to fully realize their therapeutic potential in SCI.

## 5 Conclusion

This review comprehensively elaborates on how exosomes exert practical therapeutic effects by regulating various key targets and signaling pathways after SCI. Leveraging their unique biological functions and the advantage of being amenable to engineering modification, exosomes have opened up a highly promising new approach for the repair and treatment of SCI. Although there are still challenges in standard production, targeted delivery efficiency, and the elucidation of the mechanism of action, with the deepening of research and the breakthrough of technical bottlenecks, exo therapy is expected to drive the treatment of SCI from the traditional palliative support model to a new era centered on precise regulation, promotion of nerve regeneration, and circuit remodeling.
